# Non-Destructive Detection of Chilled Mutton Freshness Using a Dual-Branch Hierarchical Spectral Feature-Aware Network

**DOI:** 10.3390/foods14081379

**Published:** 2025-04-17

**Authors:** Jixiang E, Chengjun Zhai, Xinhua Jiang, Ziyang Xu, Muqiu Wudan, Danyang Li

**Affiliations:** 1 College of Computer and Information Engineering, Inner Mongolia Agricultural University, Hohhot 010018, China; ejx@emails.imau.edu.cn (J.E.); imau_xzy@163.com (Z.X.); wudmqe@imau.edu.cn (M.W.); ldy19990125@163.com (D.L.); 2 Key Laboratory of Big Data Research and Application of Agriculture and Animal Husbandry, Hohhot 010018, China; 3 Education Examinations Authority of Inner Mongolia Autonomous Region, Hohhot 010011, China; nmgzsks@163.com

**Keywords:** hyperspectral, freshness detection, hierarchical classification, deep learning

## Abstract

Precise detection of meat freshness levels is essential for food consumer safety and real-time quality monitoring. This study aims to achieve the high-accuracy freshness detection of chilled mutton freshness by integrating hyperspectral imaging with deep learning methods. Although hyperspectral data can effectively capture changes in mutton freshness, sparse raw spectra require optimal data processing strategies to minimize redundancy. Therefore, this study employs a multi-stage data processing approach to enhance the purity of feature spectra. Meanwhile, to address issues such as overlapping feature categories, imbalanced sample distributions, and insufficient intermediate features, we propose a Dual-Branch Hierarchical Spectral Feature-Aware Network (DBHSNet) for chilled mutton freshness detection. First, at the feature interaction stage, the PBCA module addresses the drawback that global and local branches in a conventional dual-branch framework tend to perceive spectral features independently. By enabling effective information exchange and bidirectional flow between the two branches, and injecting positional information into each spectral band, the model’s awareness of sequential spectral bands is enhanced. Second, at the feature fusion stage, the task-driven MSMHA module is introduced to address the dynamics of freshness variation and the accumulation of different metabolites. By leveraging multi-head attention and cross-scale fusion, the model more effectively captures both the overall spectral variation trends and fine-grained feature details. Third, at the classification output stage, dynamic loss weighting is set according to training epochs and relative losses to balance classification performance, effectively mitigating the impact of insufficiently discriminative intermediate features. The results demonstrate that the DBHSNet enables a more precise assessment of mutton freshness, achieving up to 7.59% higher accuracy than conventional methods under the same preprocessing conditions, while maintaining superior weighted metrics. Overall, this study offers a novel approach for mutton freshness detection and provides valuable support for freshness monitoring in cold-chain meat systems.

## 1. Introduction

Mutton is a high-protein (20–23%) meat of superior quality, which is widely consumed around the globe [1], especially in regions such as Inner Mongolia in China, and New Zealand [2,3]. It serves as an essential component of daily dietary structures, supplying substantial caloric intake necessary for maintaining metabolic functions [4]. In particular, mutton contains abundant nutritional components, including essential amino acids, vitamins, and minerals, and essential fatty acids such as linoleic acid and oleic acid, which play critical roles in enhancing human immunity and preventing diseases [5,6]. However, the nutritional quality and sensory attributes of mutton are closely related to its freshness [7]. Freshness constitutes a fundamental criterion in meat quality assessment, as the degradation of freshness levels directly impacts consumer sensory perception, nutritional quality, and ultimately, market acceptance [8].

Within modern livestock practices, mutton undergoes multiple stages, including production, transportation, and retail distribution, before reaching consumers [4]. Chilled preservation and cold-chain logistics are widely adopted to meet market demands [9]. Nevertheless, the freshness of chilled mutton during these processes can be compromised by factors such as ambient temperature, humidity, and microbial contamination [10]. Specifically, prolonged storage enhances enzymatic activities and microbial growth, causing significant changes in freshness indicators such as total volatile basic nitrogen (TVB-N), total aerobic count (TAC), and most probable number (MPN) of *Escherichia coli*, thereby accelerating spoilage [11,12]. Therefore, rapid, accurate, and reliable freshness detection is critically important.

With the increasing emphasis on food safety, meat freshness detection methodologies have shifted from traditional sensory evaluations to more reliable physicochemical analyses, instrumental detection methods, and dye-based colorimetric assays [13,14,15,16]. However, physicochemical methods are often complicated, time-consuming, destructive, and costly, typically serving as quantitative benchmarks for other detection technologies. Dye-based assays remain relatively underdeveloped. In contrast, instrumental analytical techniques such as electronic noses and hyperspectral imaging have gained prominence in meat freshness assessment [17,18,19]. Electronic noses detect odor and color signals via sensors, whereas hyperspectral imaging utilizes the distinct spectral absorption and reflection properties of meat. Hyperspectral imaging, in particular, exhibits advantages such as rapidity and non-invasiveness, effectively capturing subtle changes during spoilage through correlation analyses across spectral bands and enabling precise freshness classification when integrated with data analysis models.

Currently, hyperspectral imaging combined with deep learning has been extensively applied in meat freshness research, with multidimensional prediction models constructed by integrating surface texture features or various physicochemical indicators, thereby achieving improved detection performance [20,21,22,23]. Nonetheless, due to variations in mutton sample preparation protocols and experimental conditions, spectral data are susceptible to noise interference and tend to exhibit specific distribution patterns [23,24]. Moreover, inappropriate data processing strategies may lead to substantial loss of critical spectral information [25,26,27]. Despite optimized preprocessing and feature selection techniques being available, existing models often inadequately utilize spectral band information [28,29,30].

Furthermore, many hyperspectral models still primarily rely on full-spectrum analysis, lacking dynamic perception mechanisms for spectral structure and the ability to model local feature correlations. Deep learning models typically encounter challenges such as sample dependency, feature redundancy, and class imbalance in practical applications [31,32,33]. In particular, the misclassification rate is high at ambiguous boundaries between adjacent freshness levels [34]. In this context, dual-branch network architectures have demonstrated unique advantages in spectral detection by simultaneously processing full-spectrum data and selected critical spectral bands, thus preserving macroscopic spectral trends while effectively capturing subtle compositional variations [35,36]. Although dual-branch networks incorporate cross-modal functionality and hierarchical attention mechanisms to reduce misclassification, traditional dual-branch structures suffer from insufficient inter-branch feature interactions and inappropriate class weight distributions. Particularly when handling noisy data, independent branch decisions may lead to the decoupling of discriminative cross-modal features or induce gradient conflicts during propagation, thereby causing adversarial interference between feature channels [37]. To address the above limitations, a Dual-Branch Hierarchical Spectral Feature-Aware Network (DBHSNet) was proposed, separately processing full-spectrum data and local characteristic spectral bands. Specifically, the position-enhanced bidirectional cross-attention (PBCA) module enables bidirectional feature interaction, effectively overcoming the data independence limitations inherent in traditional dual-branch architectures [38]. Then, the multi-scale enhanced multi-head attention (MSMHA) module increases the sensitivity to subtle spectral variations, enriching feature representations and reducing sample dependency. Finally, the hierarchical classification mechanism (HCM) dynamically adjusts the loss weights across different levels, resulting in improved robustness and classification balance under multi-category detection scenarios.

This study aims to reveal the variations in spectral characteristics and intrinsic quality differences of chilled mutton at varying freshness levels using hyperspectral imaging technology, and to develop an accurate freshness detection model for chilled mutton based on a dual-branch network architecture. First, hyperspectral data from chilled mutton samples over a 14-day storage period were collected and combined with freshness indicators such as TVB-N, TAC, and MPN to establish the raw freshness dataset. A multi-stage processing method was employed for preprocessing and optimal feature wavelength selection, thus enhancing the purity and quality of the dataset. Finally, a freshness evaluation framework for chilled mutton was established, accompanied by a series of comparative experiments to comprehensively assess the practical effectiveness and superiority of the DBHSNet in freshness classification tasks.

The primary contributions of this study include:

(1) Utilizing multi-indicator integrated hyperspectral data for freshness evaluation, providing biologically and chemically relevant data support.
(2) Establishing a generalized data processing strategy through optimized preprocessing and feature wavelength selection, supplemented by SHAP interpretability analyses.
(3) Introducing the PBCA module, an advanced position-encoded bidirectional cross-attention mechanism, significantly enhancing the DBHSNet’s sensitivity to long-range dependencies and fine-grained details.
(4) Developing the MSMHA module, a multi-scale enhanced multi-head attention approach, improving the DBHSNet’s capability to detect subtle freshness characteristics.
(5) Proposing the HCM module, a hierarchical classifier with dynamically optimized loss functions, effectively mitigating misclassification caused by intermediate feature ambiguity and overlapping boundaries.
(6) Designing a dual-branch spectral feature-aware network that integrates multiple modules to fully perceive spectral characteristics, achieving high-accuracy freshness detection of chilled mutton, and providing valuable insights and methodologies for future meat freshness research.

## 2. Materials and Methods

### 2.1. Preparation of Chilled Mutton Samples

Fresh mutton samples were purchased from a certified food company in the Huimin District of Hohhot, Inner Mongolia, and were obtained from 10 adult Sunit sheep slaughtered following humane methods. To ensure consistent initial conditions, the mutton samples were taken from the dorsal loin area, which is characterized by relatively uniform muscle fiber distribution [4]. To minimize the effects of air humidity and microbial contamination on freshness [39], the mutton (including skin and fat) was uniformly packaged and transported to the laboratory in a refrigerated container within approximately 1 h. In the laboratory, surface fat, fascia, and other connective tissues were removed using sterile stainless-steel knives on a standard operating bench. After 24 h of pre-cooling and aging treatment at 0–4 °C, samples were randomly divided into 6 cm × 6 cm × 1 cm pieces from three different parts, resulting in a total of 210 mutton samples.

All samples were first divided into three groups, then uniformly vacuum-packed using a vacuum packaging machine (DZ-400, Zhejiang Bespacker Machine Co., Ltd., Wenzhou, China), and continuously stored at 4 °C for 14 days. Each day, five samples were randomly selected from each group for hyperspectral data acquisition. Simultaneously, freshness indicators, including TVB-N, TAC, and MPN, were measured.

### 2.2. Acquisition of Hyperspectral Images During Chilled Storage

#### 2.2.1. Hyperspectral Imaging Equipment

The hyperspectral imaging system used in this study consisted of a Hyperspec VNIRN-Series hyperspectral imager manufactured by Headwall Photonics, a scanning platform, and two adjustable 1000 W halogen lamps. The spectral range covered by the imager was 400–1000 nm, with a spectral resolution of 2.8 nm, including a total of 750 spectral channels.

#### 2.2.2. Hyperspectral Image Acquisition

**Figure 1:**
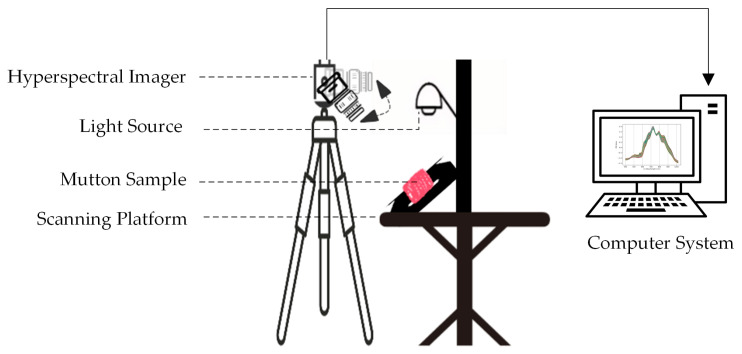
Hyperspectral data acquisition process.

The hyperspectral imaging system was preheated for 30 min before image collection, with the lens aperture adjusted to F2.0 to achieve maximum throughput. During the experiment, each sample was placed approximately 30 cm from the camera lens on the scanning platform, facing directly toward the lens [3,14]. Pixel averaging was set to six scans, and the exposure time was adjusted to maintain digital number (DN) values below 8500, avoiding signal saturation. Two 100 W halogen lamps were symmetrically placed at 45° angles approximately 35 cm from the sample. The illumination uniformity was adjusted stepwise, ensuring a uniformity greater than 90%. Hyperspectral images were then collected using the computer system. The specific acquisition process is shown in Figure 1.

Due to illumination non-uniformity and camera dark current, the spectral images were calibrated using black and white corrections, as shown in Equation (1):

(1) $$G=\frac{I-B}{W-B}$$

where I is the raw image, B is the dark calibration image, W is the white calibration image, and G is the calibrated spectral image [4]. Hyperspectral data acquisition was completed using the hyperspectral system and corresponding data acquisition software.

#### 2.2.3. Hyperspectral Data Extraction

ENVI 5.6 software was utilized to select regions of interest (ROIs) from hyperspectral images of chilled mutton samples [23]. Firstly, false-color images were constructed using wavelengths at 650 nm, 554 nm, and 553 nm, based on the RGB principle, to visually identify the tissue distributions clearly. Secondly, to avoid connective tissue interference, only lean meat regions were marked, and 20 ROIs, each sized 10 × 10 pixels, were randomly selected from each image. The DN values of all the spectral bands within these ROIs were recorded. Finally, spectral information from each ROI was compiled into an original freshness spectral dataset, resulting in a total of 4200 samples (20 × 210).

For feature selection, *Y*-values represented continuous freshness indicators, while for freshness classification, *Y*-values indicated different freshness grades.

### 2.3. Measurement of Freshness Indicators and Freshness Grading

Freshness indicators of samples were measured over a 14-day storage period. TVB-N content was determined according to GB 5009.228-2016 (National Food Safety Standard—Determination of Volatile Basic Nitrogen in Food by Semi-micro Kjeldahl Method) [40]; TAC was measured following GB 4789.2-2022 (National Food Safety Standard—Microbiological Examination of Food: Aerobic Plate Count) [41]; and MPN was enumerated based on GB 4789.3-2016 (National Food Safety Standard—Microbiological Examination of Food: Enumeration of *Escherichia coli*) [42]. Most existing studies tend to adopt binary or ternary classification schemes for freshness assessment [4,43,44,45,46,47], which may overlook transitional changes in physicochemical and microbial characteristics.

To better reflect the synergy of multiple indicators and achieve refined freshness classification, this study implemented a threshold-based cross-classification strategy based on GB 2707-2016 (National Food Safety Standard—Fresh (Frozen) Livestock and Poultry Products) and relevant literature [48]. Freshness grades were precisely defined based on the ranges of the three measured indicators [49]. Four freshness levels were established: Fresh, SubFresh, PreSpoiled, and Spoiled. The “SubFresh” category mainly represents samples with slight physicochemical changes and limited microbial proliferation, while the “PreSpoiled” category reflects cases where MPN has significantly increased even though TVB-N remains at a moderate level, suggesting that spoilage is entering a microbiologically driven mid-to-late stage. The relationships between indicator values and freshness grades are detailed in Table 1.

### 2.4. Research Methods

#### 2.4.1. Research Framework

**Figure 2:**
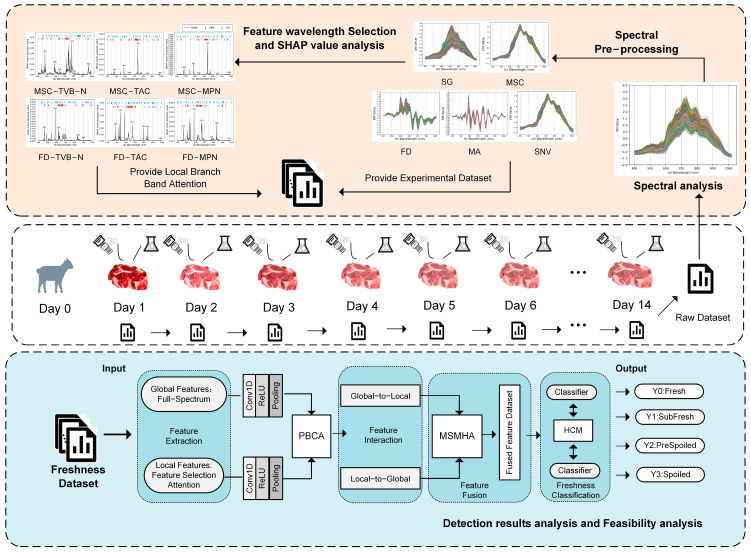
Research framework.

The workflow of this study included the following steps: (1) After preparation, mutton samples underwent freshness indicator measurements and hyperspectral image acquisition. (2) Raw spectral images were analyzed and cleaned using different preprocessing methods, with preprocessing effectiveness evaluated. (3) Regression analysis was conducted on selected spectral features, and SHAP values were employed to compare wavelength importance. (4) The original freshness data were processed using various combinations of preprocessing and feature selection methods, forming experimental datasets. (5) A Dual-Branch Hierarchical Spectral Feature-Aware Network (DBHSNet) was designed, and its detection performance was compared with CNN, LightGBM, SVM, and RF. (6) The results were evaluated and analyzed using performance metrics and confusion matrices. Specifically, as shown in the workflow of Figure 2.

#### 2.4.2. Preprocessing

The raw spectral freshness data are susceptible to noise caused by instruments, illumination conditions, and uncontrollable factors, such as sample heterogeneity and surface roughness. Therefore, preprocessing methods, including Savitzky–Golay (SG) filtering, multiplicative scatter correction (MSC), standard normal variate (SNV) transformation [50], first derivative (FD), and moving average (MA), were utilized for data cleaning [51,52]. The preprocessed dataset served as input for subsequent analyses.

These methods encompass commonly used smoothing, scatter correction, and baseline calibration approaches in hyperspectral analysis, each representing different categories such as polynomial-based smoothing and derivative-based enhancement. They offer broad applicability for handling diverse spectral variations. By evaluating the processed spectral images of each method, the preprocessed dataset served as input for subsequent analyses to demonstrate their impact on detection performance

#### 2.4.3. Feature Wavelength Selection

Feature selection significantly reduces data dimensionality in hyperspectral data analysis while retaining information highly correlated with target variables [53]. In this study, freshness indicators of chilled mutton, namely TVB-N, TAC, and MPN, were utilized as labels. The preprocessed spectral dataset obtained in Section 2.4.2 was analyzed using maximum mutual information minimization (MIM) and incremental feature selection (IFS) methods to extract spectral bands most relevant to freshness indicators. To evaluate the performance of different pre-feature-selection methods, four key regression metrics were adopted: the coefficient of determination (*R*^2^), root mean squared error (*RMSE*), mean absolute error (*MAE*), and residual prediction deviation (*RPD*). Specifically, *R*^2^ reflects the model’s goodness of fit, with higher values indicating better performance; *RMSE* and *MAE* quantify prediction errors, where lower values represent higher accuracy; and *RPD* assesses model generalization capability, with values above 3.0 signifying excellent regression performance [8,26]. Additionally, SHapley Additive exPlanations (SHAP) values were employed to further validate the significance of selected features.

1. MIM

MIM, based on mutual information theory, evaluates statistical relevance by calculating the mutual information between individual spectral bands and target variables, as shown in Equation (2) [54]. Selecting highly correlated spectral bands reflects their predictive importance for TVB-N, TAC, and MPN.

(2) I(X, Y)=H(X)+H(Y) − H(X, Y)

where *I(X, Y)* denotes the mutual information between feature *X* and target variable *Y*, *H(X)* and *H(Y)* represent the entropies of feature *X* and target variable *Y*, respectively, and *H(X, Y)* indicates their joint entropy.

2. IFS

IFS, based on joint mutual information, identifies spectral bands with high relevance and strong interactions with target variables by calculating joint information, as described in Equation (3) [55]. Unlike traditional univariate feature selection methods, IFS accounts for the synergy between features, employing combinational evaluation to reveal feature importance.

(3) I(Xi,Xj;Y)=I(Xi;Y)+I(Xj;Y)−I(Xi,Xj;Y)

where *I(Xi, Xj; Y)* represents joint mutual information between features *Xi*, *Xj*, and target variable *Y*, and *I(Xi, Y)* and *I(Xj; Y)* are mutual information between individual features *Xi*, *Xj*, and target variable *y*, respectively.

3. SHAP value validation

SHAP values, based on Shapley values from game theory, as shown in Equation (4), quantify each feature’s contribution to model predictions [28].

(4) $$\phi_{i}(f)=\sum_{S\subseteq N\backslash \{i\}}\frac{|S|!(|N|-|S|-1)!}{|N|!}\left[f(S\cup \{i\})-f(S)\right]$$

where $\phi_{i}(f)$ is the Shapley value of the *i*-th feature, *N* is the complete feature set, *S* is a subset of features, and *f(S)* represents the predicted outcome using subset *S*. SHAP contribution plots were generated to illustrate the relative importance of spectral bands and selected features for prediction [56,57]. Additionally, regression analyses were conducted on all spectral freshness feature sets to validate their predictive capabilities, identifying the optimal feature selection method.

In this study, MIM and IFS were selected as the core feature selection methods, representing univariate relevance evaluation and multivariate interaction analysis, respectively, thus offering strong representativeness and general applicability. Compared to dimensionality reduction methods such as PCA and t-SNE, this strategy avoids the interpretability loss caused by feature space transformation and helps retain the physical significance of the original wavelengths [58]. Furthermore, SHAP analysis was introduced to further enhance the interpretability and model compatibility of the selected spectral bands. To effectively evaluate the dual-branch information interaction mechanism, the selected spectral bands were utilized as local branch input to enhance freshness detection performance.

#### 2.4.4. Dual-Branch Hierarchical Spectral Feature-Aware Network

1. Feature interaction: Position-optimized bidirectional cross-attention (PBCA) module

During the feature interaction stage, traditional dual-branch methods independently process global and local features, lacking effective interaction modeling and consequently losing crucial dependency relationships [32,34]. Particularly in hyperspectral data analysis, the high dimensionality and redundancy among spectral bands make simple feature fusion approaches insufficient to capture inter-band dependencies, leading to underutilized spatial similarity and band correlation [36]. Moreover, each spectral band is sequence-sensitive, providing key contextual information via its positional arrangement in the spectrum. Directly feeding spectral data into a network without positional considerations may result in the loss of relative positional cues among bands [59].

**Figure 3:**
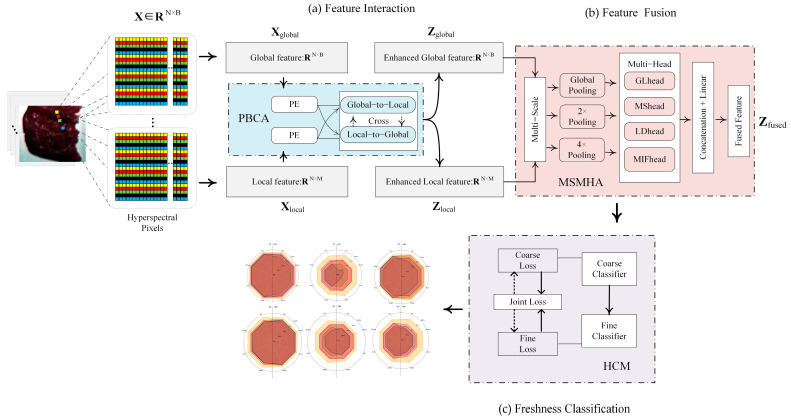
Dual-Branch Hierarchical Spectral Feature-Aware Network. (**a**) Feature Interaction, (**b**) Feature Fusion, (**c**) Freshness Classification.

Therefore, we designed the PBCA module, as shown in Figure 3a. Firstly, global and local features are separately processed through position encoding to form dynamic positional signals related to wavelength order, augmenting the learning of positional features, thus enabling the model to effectively distinguish the relative importance and sequence relationships among spectral bands [60], as represented in Equation (5).

(5) $$PE(x)=sin(x/10000^{\left(2i/d\right)}),cos(x/10000^{\left(2i+1/d\right)})$$

where *x* denotes the band index, *i* represents the position encoding dimension, and *d* indicates the total number of encoding dimensions.

Subsequently, a bidirectional cross-attention mechanism is introduced to establish a two-way information flow between global and local features, further enhancing the model’s ability to learn long-range dependencies and improving feature representation [61], as shown in Equation (6).

(6) $$\begin{array}{l} Z_{global}=\;\mathrm{Attention}(X_{global},\;X_{local},\;X_{local})\\ Z_{local}=\;\mathrm{Attention}(X_{local},\;X_{global},\;X_{global}) \end{array}$$

where *X_global_* and *X_local_* denote the input global and local features, while *Z_global_* and *Z_local_* represent the corresponding optimized features after cross-attention. Compared with conventional attention mechanisms, PBCA is better tailored to spectral data due to its explicit modeling of wavelength position and bidirectional cross-scale interactions.

2. Feature fusion: Multi-scale enhanced multi-head attention (MSMHA) module

At the feature fusion stage, many existing studies are limited to simple concatenation or weighted averaging of global and local features. However, such methods fail to capture the complex dependencies among features at different scales and may overlook subtle differences in the data. Moreover, in chilled mutton freshness detection, cross-scale correlations between spectral bands are critical for both discriminative power and robustness regarding freshness grading. Effectively capturing multi-level feature relationships can reduce the reliance on large-scale datasets [62]. Therefore, precisely modeling the complex multi-scale relationships between features has become essential for improving detection accuracy. Accordingly, we designed the MSMHA module, as shown in Figure 3b, a multi-scale enhanced multi-head attention mechanism. By integrating multi-scale pooling with multi-head attention, the MSMHA module leverages multi-scale features to model the complex relationships spanning multiple scales.

Firstly, global and local features (*Z_global_*, *Z_local_*) are pooled through a multi-scale optimization strategy to extract features of varying granularity, as illustrated in Equation (7).

(7) $$Z_{global}^{enhanced}=\mathrm{Global}\;\mathrm{Pooling}(Z_{global}),Z_{local}^{enhanced}=\mathrm{Local}\;\mathrm{Pooling}(Z_{local})$$

where $Z_{global}^{enhanced}$ and $Z_{local}^{enhanced}$ are the results of the optimized pooling of global and local features, respectively. Following multi-scale enhancement, the model captures features of varying granularity, providing abundant cross-scale information.

Next, four parallel attention heads are employed through multi-head attention mechanisms. Head 1 (Global Trend Attention Head) focuses on global trend modeling to capture overall spectral variations in freshness data. Head 2 (Local Detail Attention Head) targets specific wavelength regions strongly correlated with freshness-related chemical compositions, enhancing sensitivity to critical spectral bands. Head 3 (Long-Distance Dependency Attention Head) constructs long-range cross-band dependencies, reinforcing the understanding of nonlinear relationships among spectral bands. Head 4 (Multi-Scale Interaction Attention Head) captures cross-scale interactions, addressing potential distribution discrepancies across different feature scales. The use of four heads was empirically selected to balance complexity and interpretability.

For each head *i* (1 ≤ *i* ≤ 4), input features after multi-scale enhancement undergo computation. Linear transformations generate Query (*Q*), Key (*K*), and Value (*V*) for each head (Equation (8)).

(8) $$Q_{i}=XW_{i}^{Q},K_{i}=XW_{i}^{K},V_{i}=XW_{i}^{V}$$

where $W_{i}^{Q},W_{i}^{K}$, and $W_{i}^{V}$ are parameters for head *i* [63]. Attention force calculations for head *i* are then performed as in Equation (9):

(9) $$Head_{i}(Q_{i},K_{i},V_{i})=Softmax((Q_{i}K_{i}^{T})/sqrt(d_{k}))V_{i}$$

where *d_k_* is the dimensionality of keys, and *Softmax* normalization weights feature importance.

Finally, outputs from all the attention heads are concatenated and linearly transformed to yield fused features *Z_fused_* (Equation (10)):

(10) $$Z_{fused}=Linear(Concat(Head_{1},Head_{2},\ldots ,Head_{i}))W^{O}$$

where linear represents a fully connected layer, and *W^0^* is a linear transformation matrix mapping concatenated features into a new space. Each head focuses on different spectral characteristics, providing richer feature representation.

3. Freshness classification: Hierarchical classification mechanism (HCM) module

During the output stage, the fused features *Z_fused_* are fed into a hierarchical classifier for two-stage classification. To address overlapping category features, imbalanced sample distributions, and insufficient intermediate features in chilled mutton freshness detection, we designed the HCM module, as shown in Figure 3c. This hierarchical classifier employs a dynamic joint loss function strategy, progressively tackling classification challenges through staged optimization of coarse- and fine-grained classifiers and a dynamically balanced joint-loss design [64,65].

First, the coarse classifier partitions the samples into broad categories, simplifying the task to ensure an initial differentiation of major classes and generating intermediate features *H* (Equation (11)). The fine classifier subsequently processes these intermediate features for more nuanced classification, handling subtle inter-class differences and ultimately producing the prediction *Y* (Equation (12)).

(11) $$H=ReLU(Z_{fused}W_{shared}+b_{coarse})$$

(12) $$Y={HW}_{fine}+b_{fine}$$

where *W_shared_* and *b_coarse_* are the weight and bias parameters of the coarse classifier, whereas *W_fine_* and *b_fine_* pertain to the fine classifier.

To effectively optimize both coarse- and fine-grained classification, we employ the Cross-Entropy Loss function for each task [49], denoted as *L_coarse_* and *L_fine_* (Equation (13)).

(13) $$L_{coarse,\;fine}=CrossEntropy(y_{coarse,\;fine},Y_{coarse,\;fine})$$

where *Y_coarse_* and *Y_fine_* represent the true labels.

Moreover, to dynamically adjust the focus between coarse- and fine-grained tasks during training, a dynamic weight *w_dynamic_* is introduced. Initially, *w_dynamic_* is set to a larger value for the coarse task, then gradually shifts toward the fine task based on the number of epochs (*epoch*) and the maximum epoch (*epoch_ma_*_x_), as in Equation (14). Concurrently, *w_dynamic_* is adjusted according to the relative losses at each stage (Equation (15)).

(14) $$w_{dynamic}=epoch/{epoch}_{max}$$

(15) $$w_{dynamic}=L_{coarse}/(L_{coarse}+L_{fine})$$

Using this dynamic loss optimization strategy, the model automatically updates *w_dynamic_* across various training stages, leading to the total loss function *L_total_* in the HCM module (Equation (16)). This allows the model to focus on the most critical tasks or objectives at each phase, accelerating convergence and enhancing overall performance.

(16) $$L_{total}=w_{dynamic}L_{coarse}+(1-w_{dynamic})L_{fine}$$

The DBHSNet adopts a modular architecture, allowing flexible substitution or integration of external components. This design facilitates future extensions, such as multi-modal fusion or the incorporation of task-specific classifiers.

#### 2.4.5. Evaluation of Models

In the preliminary experiments, four key metrics were used to evaluate the model performance under different feature selection methods: the coefficient of determination (*R*^2^), root mean squared error (*RMSE*), mean absolute error (*MAE*), and residual prediction deviation (*RPD*). *R*^2^ measures the model’s goodness of fit, where a higher *R*^2^ indicates better performance. *RMSE* and *MAE* quantify prediction errors, with smaller values implying higher accuracy. RPD evaluates the model’s generalization capability, where values exceeding 3.0 indicate excellent regression performance.

In subsequent experiments, to meet the high-accuracy requirements of mutton freshness detection, we employed *Accuracy*, *Weighted Precision*, *Weighted Recall*, and *Weighted F1-score* as the evaluation metrics, as shown in Equations (17)–(20). To ensure the robustness of the model evaluation, a 5-fold cross-validation strategy was employed to minimize the influence of random data partitioning.

Let the number of classes be *n*, with *N_i_* samples in the *i*-th class and a total of *N* samples overall. In the classification tasks performed in this study, the effective positive (*EP_i_*) denotes the number of positive samples correctly identified; the undetected positive (*UP_i_*) refers to positive samples mistakenly classified as negative; the false alert positive (*FAP_i_*) indicates negative samples incorrectly classified as positive; and the effective negative (*EN_i_*) denotes the number of negative samples correctly identified.

(17) $$Accuracy=\frac{EP+EN}{EP+FAP+EN+UP}$$

(18) $$Weighted\;Recall=\sum_{i=1}^{n}\left(\frac{Ni}{N}\times \frac{EPi}{EPi+UPi}\right)$$

(19) $$Weighted\;Precision=\sum_{i=1}^{n}\left(\frac{Ni}{N}\times \frac{EPi}{EPi+FAPi}\right)$$

(20) $$Weighted\;F1\;Score=2\times \frac{Weighted\;Precision\times Weighted\;Recall}{Weighted\;Precision+Weighted\;Recall}$$

### 2.5. Implementation

The experimental platform is configured with an Intel Core i5-10300H CPU (2.50 GHz), 16 GB of RAM, and Nvidia RTX 1650Ti GPU, running on Windows 10 (Version 22H2, OS Build 19045.5487, 64-bit). Python 3.11 was used for coding, and Pycharm was employed for model development and hyperparameter tuning.

## 3. Results and Discussion

### 3.1. Analysis of Freshness Indicator Metrics

**Figure 4:**
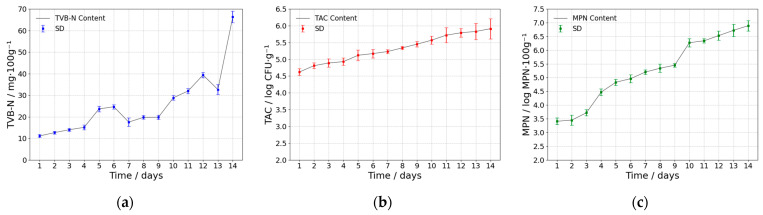
Freshness indicator content curves: (**a**) TVB-N, (**b**) TAC, (**c**) MPN.

Figure 4 illustrates the variations in freshness indicators over a 14-day storage period. During storage, TVB-N, TAC, and MPN levels all gradually increased, but at different stages and with varying rates.

TVB-N rose from 6 mg·100 g^−1^ on Day 1 to 9 mg·100 g^−1^ on Day 3, indicating that protein degradation remained in its initial phase and the mutton retained a relatively stable freshness [24]. Subsequently, on Days 4 and 10, MPN and TVB-N levels exhibited significant increases, aligning with rapid microbial proliferation and intensified enzymatic protein degradation. After Day 10, the accelerated accumulation of microbial metabolites further accelerated the spoilage process, signifying a more advanced stage of deterioration [17]. Moreover, due to the involvement of non-protein nitrogen sources in complex biochemical reactions within the tissue, a transient decline in TVB-N may occur when the consumption of nitrogenous compounds exceeds their production. Consequently, during Days 5–6 and 7–9, TVB-N levels exhibited temporary plateaus, likely due to a transient balance between the production and consumption of nitrogenous substances [4,66].

Meanwhile, TAC rose steadily from an initial 4.4 log CFU·g^−1^ to about 6.1 log CFU·g^−1^, suggesting that low temperatures partially inhibited aerobic bacterial growth but did not prevent overall accumulation [23]. In contrast, MPN increased only by 0.3 log MPN·100 g⁻^1^ during the early storage stage, yet after Day 4, it surged notably due to the rapid onset of microbial decomposition and enzymatic reactions in the mid-to-late storage period.

Overall, protein decomposition and the accumulation of volatile alkaline substances progressed slowly in early storage, resulting in modest increases in TVB-N and MPN. However, as storage continued and microbial metabolic activity and enzymatic reactions intensified, meat spoilage accelerated, causing sharp rises in TVB-N and MPN on Days 4 and 10, whereas TAC maintained a gradual upward trend [24]. By the end of Day 14, TVB-N content had increased nearly 10-fold compared to initial levels, and MPN surpassed 7.0 log MPN·100 g^−1^, indicating severe spoilage of the mutton. Although low-temperature storage can partially delay the growth of aerobic bacteria and other microorganisms, it cannot effectively halt the rapid surge in late-stage microbial activity. Notably, these parameters may be affected by temperature and airborne microorganisms, highlighting the necessity of strict environmental control during the experiment. These findings provide a valuable reference for subsequent dynamic freshness monitoring and detection model development.

### 3.2. Data Preprocessing Results Analysis and Comparison

In this study, to more accurately represent the spectral information of chilled mutton and enhance the classification performance for freshness detection, we applied various preprocessing techniques to the raw spectral data, including MSC, FD, SNV, SG, and MA.

**Figure 5:**
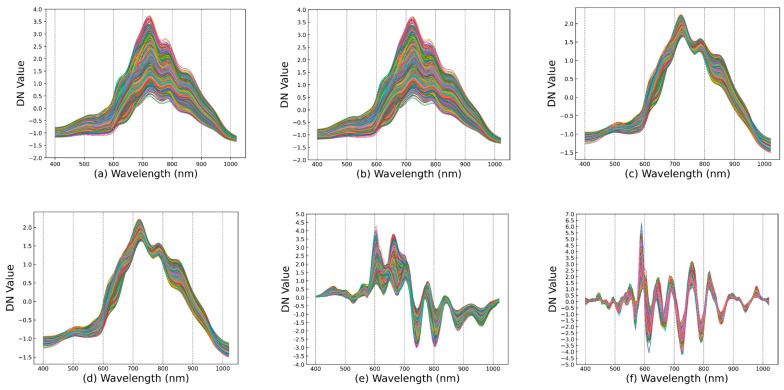
Preprocessing results: (**a**) Raw, (**b**), SG (**c**), MSC (**d**), SNV (**e**), FD, (**f**) MA.

Figure 5 presents the spectral images obtained after applying various preprocessing methods. As shown in Figure 5c,d, MSC and SNV effectively mitigated scattering deviations due to granularity and density differences caused by sample slicing, thereby aligning the spectral baselines via standardization or linear regression calibration [28,67]. However, both methods are relatively sensitive to outliers, and the presence of strong noise may compromise the overall baseline correction performance. Meanwhile, Figure 5e demonstrates that FD effectively highlights subtle peaks and valleys while reducing baseline drift [68]. This enhanced expression of local features contributes to the extraction of freshness-related information and is particularly beneficial for capturing early-stage spectral changes during meat spoilage. Figure 5b,f indicate that both SG and MA can effectively suppress high-frequency noise and smooth the spectral curves [51]. Nevertheless, they differ slightly in preserving fine peak features: SG retains sharp peaks due to its local polynomial fitting, while MA tends to blunt sharper signals, which may lead to partial loss of spectral detail.

Overall, each preprocessing method offers unique advantages in noise suppression, baseline correction, and peak/valley enhancement. Appropriately integrating these methods can further improve the quality and interpretability of spectral data. Therefore, the preprocessed dataset is directly employed as input for feature selection and detection models, aiming to identify an optimal combination of data processing strategies and higher-efficiency data inputs for subsequent quantitative and qualitative analyses.

### 3.3. Analysis of Feature Selection

Table 2 compares the predictive performance of MIM and IFS under different preprocessing methods for freshness indicators. We evaluated each combination using regression metrics (*R*^2^, *RMSE*, *MAE*, and *RPD*) and verified the contribution of selected spectral bands via SHAP values. The results indicate that feature band selection exerts a noticeable impact on both preprocessing methods and predictive accuracy.

First, regarding predictive outcomes, the use of MIM or IFS significantly boosted model performance. When building full-spectrum models for TVB-N, TAC, and MPN with MSC, SNV, or FD preprocessing, the key bands selected by MIM and IFS both exhibited higher predictive precision, indicating their effectiveness in capturing critical freshness-related wavelengths. Particularly under the FD-IFS combination, TVB-N achieved an *R²* of 0.8757 and an *RMSE* of 0.3601, surpassing other methods or the raw data with the lowest error metrics; furthermore, TAC and MPN predictions yielded *RPD* > 3.5, indicating strong model stability and robust feature selection outcomes.

**Figure 6:**
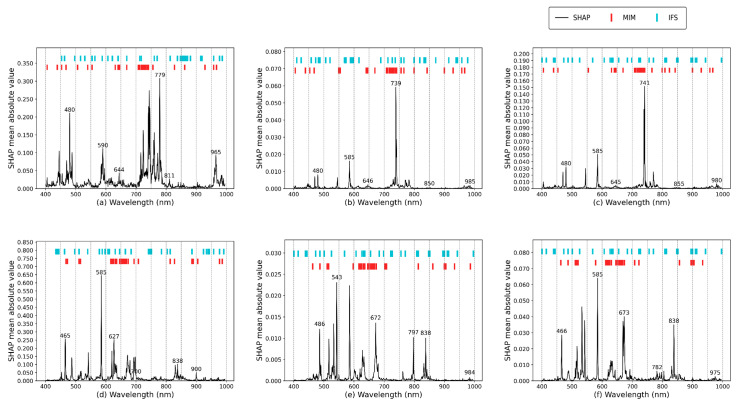
Distribution and contribution comparison of feature wavelengths under different indicators: (**a**) MSC-TVB-N, (**b**) MSC-TAC, (**c**) MSC-MPN, (**d**) FD-TVB-N, (**e**) FD-TAC, (**f**) FD-MPN.

Nonetheless, no single combination demonstrated uniformly high efficiency. Specifically, SG-MIM and MA-MIM performed poorly, as revealed by the spectral band distributions in Figure 6. Possibly, MIM overlooked certain band combinations strongly correlated with changes in freshness indicators, biasing the model toward highly contributive wavelengths and leading to higher regression errors in these two pairings [28]. Consequently, IFS exhibits more stable performance for high-dimensional data with complex inter-band correlations, whereas MIM is more suited to the fine-grained selection of highly contributive spectral bands. From a domain-specific perspective, while techniques such as PCA or autoencoders might further reduce dimensional complexity, they often obscure physically meaningful wavelength interpretations, which is crucial for subsequent biological or chemical analysis [58]. By retaining the original spectral bands, MIM and IFS offer a more transparent feature selection process, aligning closely with the domain-specific requirements of meat freshness detection.

Figure 6 compares the distributions and contributions of the selected feature bands for the three freshness indicators under optimal and suboptimal data combinations, highlighting how different selection strategies emphasize distinct spectral regions. Certain critical bands make prominent contributions to predicting freshness indicators, while the similarity between MIM- and IFS-chosen bands and the high-contribution regions in the SHAP plots is also validated. On one hand, the MIM-selected bands largely cluster at 450–585 nm and 600–750 nm. Among them, the 480–550 nm range shows high SHAP values, implying potential interactions with pigment-derived antioxidants and microbial coenzymes. Additionally, the 438 nm and 462 nm bands relate to fatty acid absorption, indicating the degree of fat oxidation in meat [69], while 515 nm, 683 nm, and 688 nm concern transitions of iron ions in myoglobin and heme proteins, reflecting protein decomposition states and thereby freshness changes [14]. The 818 nm and 861 nm bands capture water absorption characteristics [46]. On the other hand, the IFS-selected bands exhibit a more dispersed distribution but align better with SHAP peaks. In TAC and MPN prediction, bands at 585 nm, 739 nm, and 838 nm display notably high contributions, illustrating the close link between feature selection, chemical composition, and microbial metabolic processes. Beyond 900 nm, both selection strategies yield fewer selected bands, consistent with the diminished abundance of indicator compounds relevant to freshness at longer wavelengths.

Overall, preprocessing combined with feature selection significantly enhances the predictive accuracy of hyperspectral models, optimizes computational efficiency, and improves the robustness and interpretability of local branches. These chosen key bands provide feasible inputs to subsequent detection networks, offering precise feature information that enables more efficient local network focus and delivering reliable spectral support for meat freshness assessment tasks.

### 3.4. Analysis of Model Detection Results

To verify the effectiveness of the DBHSNet in chilled mutton freshness detection, we conducted ablation experiments and compared classification metrics with typical algorithmic models. We also generated confusion matrices to illustrate the detection outcomes. For this study, the experimental dataset was approximately divided into training, validation, and test sets in a 4:1:1 ratio to maintain a balanced distribution for model evaluation.

#### 3.4.1. Freshness Classification Results of Chilled Mutton

We established a performance evaluation framework for CNN, SVM [70], LightGBM [71], RF, and the DBHSNet to ensure the accuracy of mutton freshness detection; the specific results are shown in Table 3. The fully preprocessed hyperspectral dataset of chilled mutton was utilized as the DBHSNet input, while spectral bands derived from various combination methods were assigned to the local branch.

According to Table 3, the DBHSNet model achieved the best performance across all metrics under the FD-IFS combination. On one hand, its *accuracy* reached 99.72%, outperforming the next-best model (LightGBM) by 0.97%, and the *weighted F1-score* exceeded 99.50%, indicating robust stability for multi-class scenarios and imbalanced data. On the other hand, the DBHSNet model’s *weighted precision* and *weighted recall* both surpassed 99.00%, suggesting highly efficient use of intermediate features to distinguish freshness levels. Notably, under identical preprocessing conditions, the DBHSNet achieved a maximum accuracy improvement of 7.59% compared to other models, with the most pronounced advantages observed under the MA and Raw combinations. This suggests that the model maintains robust discriminative capacity even in the presence of noisy or low-quality spectral data while also demonstrating superior feature extraction and generalization capabilities across diverse data conditions. From a food safety perspective, the DBHSNet could help prevent spoiled meat from entering the market, thereby mitigating health risks and economic losses.

Furthermore, examining different preprocessing methods, FD and MSC each enhanced model performance by extracting frequency-domain features and correcting scattering noise while reinforcing the stability of chemical characteristics, thus making a substantial contribution to freshness-related feature extraction [51]. In contrast, MA effectively smoothed noise but also led to the loss of certain spoilage signals, reflecting its limitations in freshness detection. Moreover, under FD and MSC, CNN, LightGBM, and RF also exhibited robust performance, indicating these two preprocessing strategies are particularly well-suited for dealing with noise and redundancy in raw spectral data.

Compared to traditional spectroscopic techniques such as near-infrared or Raman spectroscopy, which typically cover limited spectral ranges and are primarily sensitive to specific molecular vibrations, hyperspectral imaging provides broader spectral coverage and richer feature representations. This allows the DBHSNet to capture more subtle spectral variations related to mutton freshness, enabling efficient and accurate freshness classification. Such capability is particularly valuable for food safety applications that require rapid, non-destructive, and comprehensive quality assessment, and holds potential for extension to other types of meat.

#### 3.4.2. Ablation Study

Table 4 summarizes the ablation study findings for the DBHSNet under varying local feature attention configurations.

From Table 4, we observe a pronounced nonlinear performance gain as each module is progressively introduced and compared. First, the HCM module alone offers fundamental optimization. When HCM is enabled independently, *accuracy* increases significantly across all three experimental groups: by 3.33% in FD-MIM, 2.36% in FD-IFS, and 2.92% in MSC-MIM. Moreover, the difference between the *weighted F1-score* and *accuracy* remains below 0.07%, indicating that HCM effectively enhances inter-class discriminative power for freshness features. Second, in the FD-MIM group, combining PBCA + HCM outperforms MSMHA + HCM by 2.08%, whereas adding MSMHA to form the full-module configuration boosts this margin to 5.83%.

These superimposed results demonstrate that the hierarchical classification mechanism built by HCM effectively exploits the multi-scale feature fusion from MSMHA. Notably, misclassification cases in intermediate freshness levels were reduced more substantially when all three modules were combined, suggesting that the HCM can better handle ambiguous boundaries by dynamically reweighting decision layers. However, each module combination exhibits distinct methodological advantages, so practical detection applications should adopt tailored strategies based on data characteristics. Third, the fully integrated modules achieved peak performance in freshness detection. The FD-IFS group’s *accuracy* improved by 11.80% relative to the baseline, while metric fluctuations remained below 1%, indicating a positive coupling between FD-IFS and the model’s spectral feature awareness. When all modules are enabled, both FD-MIM and FD-IFS groups yield weighted metrics above 99.00%. In addition to accuracy improvements, the full-module configuration also exhibited enhanced computational efficiency, reducing inference time and indicating strong potential for real-time deployment in chilled meat quality monitoring workflows.

Hence, the integration of PBCA, MSMHA, and HCM significantly boosts model performance and feature representation, offering high robustness for real-time chilled mutton quality monitoring. Overall, the ablation study not only validates the contribution of each proposed module to freshness detection but also provides insights into the modular adaptability of the DBHSNet under varying data quality scenarios.

#### 3.4.3. Comparison of Classification Performance

**Figure 7:**
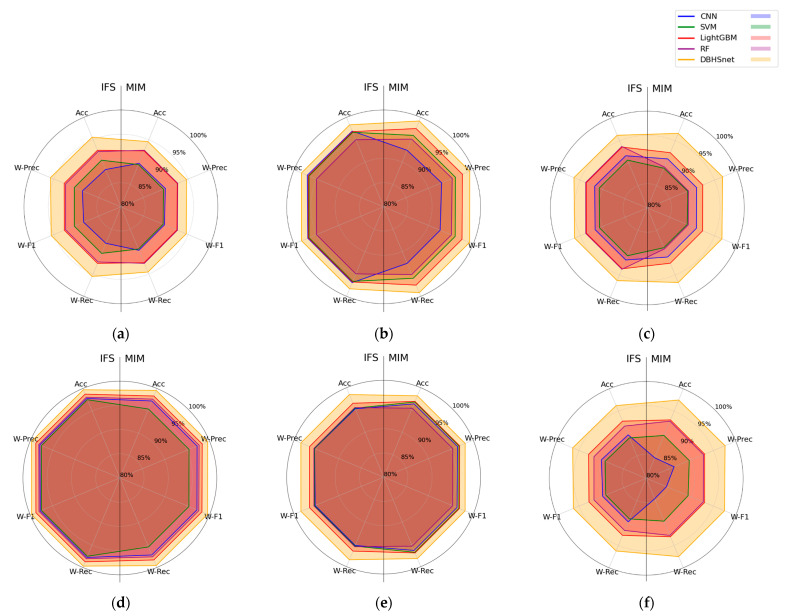
Comparison of classification performance across different models under various combination methods: (**a**) Raw, (**b**) SNV, (**c**) SG, (**d**) FD, (**e**) MSC, (**f**) MA.

To further verify the superiority of the DBHSNet in freshness detection, we modeled various algorithms under identical preprocessing conditions to assess the impact of different preprocessing–feature selection strategies on model performance, as illustrated in Figure 7.

Using a performance radar chart for multidimensional comparisons reveals that, under various preprocessing methods, the DBHSNet exhibits competitive results in terms of classification *accuracy*, *weighted precision*, *weighted recall*, and *weighted F1 Score*. Notably, it maintains a balanced profile across these metrics, indicating that the model retains its performance advantage when facing different data processing strategies—particularly under data imbalance—thereby underscoring the DBHSNet’s flexibility and extensive applicability in complex tasks.

Compared with traditional dual-branch networks [31,35], the proposed DBHSNet alters the independent contributions of global and local branches. Through PBCA, it enables bidirectional information flow between global and local features and injects positional information into each band, emphasizing collaborative changes among consecutive bands. In addition, to address the overall spoilage process and transient release of varied metabolites, MSMHA exploits a task-driven multi-head attention design with cross-scale fusion to better capture global spectral trends and fine-grained details. During classification, dynamic weights are assigned based on training epochs and relative losses to balance the hierarchical classifiers; HCM consequently reduces overlapping effects among intermediate features. These improvements culminate in a performance breakthrough for chilled mutton freshness detection.

#### 3.4.4. Confusion Matrix

**Figure 8:**
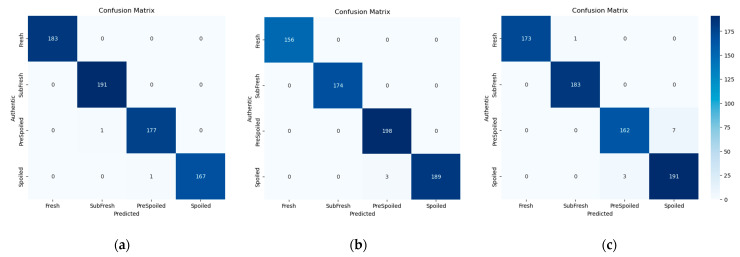
Confusion matrices: (**a**) FD-IFS, (**b**) FD-MIM, (**c**) MSC-MIM.

Figure 8 displays the confusion matrices for the DBHSNet under three different data processing combinations. Each confusion matrix highlights the relationship between true and predicted labels across various classes.

With FD-IFS for the local branch, the DBHSNet incurred no misclassifications for “Fresh” or “Spoiled” samples, and only one error each for “SubFresh” and “PreSpoiled.” Under FD-MIM, the DBHSNet similarly achieved high accuracy, with minimal misclassification of fresh and spoiled samples; out of 201 “PreSpoiled” samples, 198 were correctly identified, and only 3 were wrongly classified as spoiled. Although the majority of samples were correctly classified, the MSC-MIM approach yielded more misclassifications, particularly among adjacent freshness levels. This may be attributed to overlapping intermediary features during the freshness transition stage, influencing classification outcomes.

Thus, compared to other studies [4,43,46], the confusion matrices further validate the DBHSNet’s superiority in mutton freshness detection, although challenges persist when distinguishing subtle differences between classes. Moreover, from a cost-benefit perspective, the DBHSNet offers significant practical value. First, by substantially reducing misclassification—especially in ambiguous intermediate categories—it lowers the risk of mistakenly accepting spoiled meat, thereby minimizing potential contamination and public health threats. Second, improved classification precision reduces the likelihood of repeated testing and product recalls, resulting in considerable savings in labor, time, and reagent costs compared to traditional methods.

Future work could integrate multi-modal sensor data to enrich freshness-related feature inputs, thereby further improving classification robustness in transitional stages and facilitating the engineering application of chilled meat monitoring technologies.

## 4. Conclusions

This study employed multi-indicator integrated hyperspectral data to evaluate the freshness of chilled mutton, subdividing the samples into four distinct freshness levels. First, by optimizing preprocessing and feature selection methods and comparing spectral band contributions using SHAP values, we established a generally optimal data processing strategy for subsequent research. Next, the DBHSNet was proposed, leveraging multiple integrated modules to fully capture the spectral characteristics of mutton and thereby achieve high-accuracy detection of chilled mutton freshness.

Among all the tested models, FD-IFS and MSC-MIM emerged as the optimal and suboptimal data processing approaches, respectively, while the DBHSNet attained the best classification metrics, with accuracy and weighted classification indices both exceeding 99.00%. Furthermore, the model exhibited only a small number of misclassifications, primarily occurring between adjacent freshness levels, underscoring the critical role of intermediate features in classification decisions. Overall, the DBHSNet model constructed herein meets practical requirements for chilled mutton freshness detection and significantly enhances the representation efficiency of freshness-related features in hyperspectral data. Future work will explore the integration of hyperspectral imaging with gas sensors or fluorescence spectroscopy on the same batch of samples to further improve detection accuracy and model generalizability. Additionally, batch-wise spectral acquisition systems combined with detection models will be developed for real-time deployment, supporting rapid-response requirements in industrial quality control workflows.

## Figures and Tables

**Table 1:** Freshness Grade Classification.

TVB-N/mg·100 g^−1^	TAC/log CFU·g^−1^	MPN/log MPN·100 g^−1^	Label
(0, 15]	≤6.0	≤5.0	Fresh
(15, 25]	≤6.0	≤5.0	SubFresh
(15, 25]	≤6.0	>5.0	PreSpoiled
>25	>6.0	>5.0	Spoiled

**Table 2:** Comparison of prediction performance of feature selection under different preprocessing methods.

Preprocessing Methods	Metric	MIM				IFS			
		R²	RMSE	MAE	RPD	R²	RMSE	MAE	RPD
RAW	TVB-N	0.6972	0.5621	0.3862	1.8171	0.5548	0.6815	0.4273	1.4988
	TAC	0.7098	0.5538	0.3502	1.8563	0.7291	0.535	0.3358	1.9214
	MPN	0.7599	0.4974	0.3318	2.0409	0.8110	0.4413	0.2939	2.3004
MSC	TVB-N	0.8399	0.4087	0.2948	2.4992	0.7939	0.4637	0.3066	2.2028
	TAC	0.8891	0.3424	0.2353	3.0028	0.9019	0.3219	0.2214	3.1932
	MPN	0.8852	0.3439	0.2377	2.9520	0.8908	0.3355	0.2181	3.0257
MA	TVB-N	0.5978	0.6477	0.4312	1.5768	0.6633	0.5926	0.3956	1.7233
	TAC	0.7125	0.5512	0.3688	1.8650	0.7311	0.5331	0.3385	1.9283
	MPN	0.7697	0.4872	0.3297	2.0838	0.8114	0.4409	0.2944	2.3029
SNV	TVB-N	0.8177	0.4361	0.3070	2.3421	0.8414	0.4067	0.2867	2.5110
	TAC	0.8888	0.3428	0.2365	2.9985	0.8917	0.3383	0.2333	3.0389
	MPN	0.9001	0.3209	0.2204	3.1638	0.8900	0.3367	0.2188	3.0154
FD	TVB-N	0.7586	0.5018	0.3239	2.0355	0.8757	0.3601	0.2563	2.8364
	TAC	0.8336	0.4194	0.2459	2.4512	0.9261	0.2795	0.2114	3.6785
	MPN	0.9024	0.3172	0.2038	3.2009	0.9387	0.2514	0.1847	4.0378
SG	TVB-N	0.5957	0.6494	0.4282	1.5727	0.6656	0.5906	0.3955	1.7293
	TAC	0.7479	0.5161	0.3475	1.9917	0.6845	0.5774	0.3552	1.7804
	MPN	0.7584	0.4990	0.3479	2.0346	0.8121	0.4401	0.2931	2.3069

**Table 3:** Classification metrics of different models under various preprocessing–feature selection combinations.

Model	Preprocessing	MIM				IFS			
		*Accuracy* */%*	*Weighted Precision* */%*	*Weighted Recall* */%*	*Weighted F1 Score* */%*	*Accuracy* */%*	*Weighted Precision* */%*	*Weighted Recall* */%*	*Weighted F1 Score* */%*
CNN	Raw	89.72	89.91	89.72	89.72	88.33	88.60	88.33	88.15
	MSC	92.64	92.95	92.64	92.62	96.94	97.05	96.94	96.95
	FD	97.22	97.25	97.22	97.22	97.78	97.87	97.78	97.78
	SG	90.97	91.00	90.97	90.97	91.67	91.74	91.67	91.63
	SNV	96.39	96.52	96.39	96.39	95.28	95.42	95.28	95.27
	MA	89.91	89.27	89.58	89.44	89.72	90.00	89.72	89.72
SVM	Raw	89.44	89.49	89.44	89.42	90.42	90.41	90.42	90.40
	MSC	95.97	96.01	95.97	95.97	96.67	96.67	96.67	96.67
	FD	95.42	95.45	95.42	95.42	95.50	97.51	97.50	97.50
	SG	88.89	89.03	88.89	88.90	90.69	90.74	90.69	90.68
	SNV	96.81	96.83	96.81	96.81	95.42	95.44	95.42	95.41
	MA	89.58	89.61	89.58	89.57	89.03	89.18	89.03	89.07
LightGBM	Raw	92.50	92.59	92.50	92.49	92.64	92.67	92.64	92.64
	MSC	97.50	97.59	97.50	97.50	96.81	96.81	96.81	96.80
	FD	98.33	98.39	98.33	98.33	98.75	98.77	98.75	98.75
	SG	92.36	92.38	92.36	92.35	93.61	93.63	93.61	93.60
	SNV	96.94	96.97	96.94	96.94	96.53	96.53	96.53	96.53
	MA	93.06	93.06	93.06	93.05	92.78	92.88	92.78	92.76
RF	Raw	92.64	92.69	92.64	92.62	92.36	92.36	92.36	92.33
	MSC	95.14	95.31	95.14	95.13	95.00	95.00	95.00	94.99
	FD	97.64	97.70	97.64	97.64	98.06	98.11	98.06	98.06
	SG	89.17	89.17	89.17	89.14	93.75	93.76	93.75	93.71
	SNV	95.42	95.44	95.42	95.42	95.56	95.57	95.56	95.55
	MA	92.78	92.78	92.78	92.75	91.67	91.67	91.67	91.66
DBHSNet	Raw	94.58	94.66	94.58	94.58	95.56	95.74	95.56	95.57
	MSC	98.48	98.48	98.47	98.47	98.06	98.09	98.06	98.06
	FD	99.58	99.61	99.59	99.58	99.72	99.73	99.72	99.71
	SG	96.67	96.81	96.67	96.66	96.25	96.38	96.25	96.25
	SNV	98.19	98.20	98.19	98.19	98.33	98.34	98.33	98.33
	MA	97.50	97.60	97.50	97.49	96.25	96.42	96.25	96.25

**Table 4:** Ablation study results of the DBHSNet.

Local data	PBCA	MSMHA	HCM	*Accuracy* */%*	*Weighted* *Precision* */%*	*Weighted* *Recall* */%*	*Weighted* *F1 Score* */%*
FD-MIM	**✗**	**✗**	**✗**	86.53	86.81	86.53	86.44
	**✗**	**✗**	**✓**	89.86	89.92	89.86	89.87
	**✗**	**✓**	**✓**	91.67	91.65	91.67	91.66
	**✓**	**✗**	**✓**	93.75	93.75	93.75	93.75
	**✓**	**✓**	**✗**	96.53	96.57	96.52	96.55
	**✓**	**✓**	**✓**	99.58	99.61	99.59	99.58
FD-IFS	**✗**	**✗**	**✗**	87.92	88.06	87.92	87.91
	**✗**	**✗**	**✓**	90.28	90.41	90.28	90.26
	**✗**	**✓**	**✓**	93.75	93.76	93.75	93.74
	**✓**	**✗**	**✓**	95.28	95.3	95.28	95.27
	**✓**	**✓**	**✗**	97.22	97.22	97.25	97.23
	**✓**	**✓**	**✓**	99.72	99.73	99.72	99.71
MSC-MIM	**✗**	**✗**	**✗**	86.39	86.39	86.62	86.36
	**✗**	**✗**	**✓**	89.31	89.28	89.31	89.20
	**✗**	**✓**	**✓**	92.92	92.91	92.92	92.91
	**✓**	**✗**	**✓**	94.17	94.30	94.17	94.18
	**✓**	**✓**	**✗**	96.25	96.26	96.25	96.25
	**✓**	**✓**	**✓**	98.48	98.48	98.47	98.47

Note: “**✓**” indicates the presence of the module; “**✗**” indicates its absence.

## Data Availability

The original contributions presented in this study are included in the article. The hyperspectral data submitted do not contain farm or processor-identifiable information. Further inquiries can be directed to the corresponding author/s.
